# Positive impact of low-dose, high-energy radiation on bone in partial- and/or full-weightbearing mice

**DOI:** 10.1038/s41526-019-0074-3

**Published:** 2019-06-04

**Authors:** Rihana S. Bokhari, Corinne E. Metzger, Jeremy M. Black, Katherine A. Franklin, Ramon D. Boudreaux, Matthew R. Allen, Brandon R. Macias, Harry A. Hogan, Leslie A. Braby, Susan A. Bloomfield

**Affiliations:** 1 Health and Kinesiology, Texas A&M University, College Station, TX USA; 2 Mechanical Engineering, Texas A&M University, College Station, TX USA; 3 Biomedical Engineering, Texas A&M University, College Station, TX USA; 4 Anatomy & Cell Biology, Indiana University School of Medicine, Indianapolis, IN USA; 5 KBRwyle, Cardiovascular and Vision Laboratory, NASA Johnson Space Center, Houston, TX USA; 6 Nuclear Engineering, Texas A&M University, College Station, TX USA

**Keywords:** Physiology, Structural biology

## Abstract

Astronauts traveling beyond low Earth orbit will be exposed to galactic cosmic radiation (GCR); understanding how high energy ionizing radiation modifies the bone response to mechanical unloading is important to assuring crew health. To investigate this, we exposed 4-mo-old female Balb/cBYJ mice to an acute space-relevant dose of 0.5 Gy ^56^Fe or sham (*n* = ~8/group); 4 days later, half of the mice were also subjected to a ground-based analog for 1/6 g (partial weightbearing) (G/6) for 21 days. Microcomputed tomography (µ-CT) of the distal femur reveals that ^56^Fe exposure resulted in 65–78% greater volume and improved microarchitecture of cancellous bone after 21 d compared to sham controls. Radiation also leads to significant increases in three measures of energy absorption at the mid-shaft femur and an increase in stiffness of the L4 vertebra. No significant effects of radiation on bone formation indices are detected; however, G/6 leads to reduced % mineralizing surface on the inner mid-tibial bone surface. In separate groups allowed 21 days of weightbearing recovery from G/6 and/or ^56^Fe exposure, radiation-exposed mice still exhibit greater bone mass and improved microarchitecture vs. sham control. However, femoral bone energy absorption values are no longer higher in the ^56^Fe-exposed WB mice vs. sham controls. We provide evidence for persistent positive impacts of high-LET radiation exposure preceding a period of full or partial weightbearing on bone mass and microarchitecture in the distal femur and, for full weightbearing mice only and more transiently, cortical bone energy absorption values.

## Introduction

Bone is sensitive to altered mechanical loading; spaceflight induces significant losses in mass and structural integrity. Low-bone quality in astronauts upon returning to Earth or landing on Mars is an area of high priority to solve prior to exploration class missions due to an increased risk of fracture, which could be detrimental to a long-duration mission outside of low earth orbit.^1^ Due to the minimal medical capabilities of future extra-planetary missions it is possible that a broken bone could become a serious health risk.^2^ In-flight studies and those using ground-based analogs in humans and rodents have shown bone loss to be an important risk faced by astronauts if effective countermeasures are not employed.^1,3–6^ Whether exercise equipment enabling high intensity resistance training will be available on exploration class missions is yet to be determined. On those long-duration missions, astronauts will not only spend months in microgravity during transit but also extensive time in partial gravity. It is, therefore, important to understand how partial mechanical loading, such as Mars’ one-third and the Lunar one-sixth gravity environments, may affect bone mass and mechanical integrity.

Of further concern are the effects of space radiation, which could worsen the deleterious effects of spaceflight on the skeletal system. On future missions outside of low Earth orbit astronauts will encounter Galactic Cosmic Radiation (GCR), high-energy ionizing radiation which is present at extremely low, but constant, doses.^7^ Due to the difficulty, expense, and dangers of experimentation outside of low-Earth orbit, experiments investigating GCR relevant radiation influence on bone must be done using ground-based analogs. To date, most studies involving radiation exposure in animals have utilized acute exposures at doses of greater than 1 Gray (Gy) even though the total dose expected on Moon and Mars missions is below 1 Gy.^7^ Importantly, there is limited evidence thus far for the interactive effects of radiation exposure and reduced weightbearing.

One commonly used ground analog model for microgravity is rodent hindlimb suspension; however, this cannot simulate the partial weightbearing environment of the Moon and Mars.^6^ Previously, we and others have investigated the impact of partial weightbearing on murine bone, demonstrating that bone quantity and quality decrease even with partial unloading of the hindlimbs.^8–12^ This study aims to investigate the bone response to, and ability to recover from, low-dose, high-linear energy transfer (LET) radiation exposure combined with partial weightbearing simulating the 1/6 g of the Lunar environment. Our hypotheses were, first, that exposure to an acute 0.5 Gy dose of ^56^Fe would exacerbate the negative impact of 21 days of partial weightbearing at one-sixth total body weight (G/6) on cancellous bone mass and microarchitecture, bone mechanical properties and bone formation indices. Secondly, we hypothesized that there would be a sustained, long-term negative impact on trabecular bone mass, bone mechanical properties, and bone formation in response to low-dose, high-LET radiation and/or partial weightbearing as measured after 21 days of normal weightbearing recovery.

## Results

### Body mass changes due to PWB and radiation were recovered to control levels after 21 days

Animals were divided into four groups: WB-Sham, WB-Rad, G/6-Sham, and G/6-Rad. At the start of the study body mass was not different among groups (22.55 ± 1.38 g for all mice at 16 weeks of age). After 21 days of G/6, body mass was 11% lower in G/6 groups compared to WB groups (Table 1; Kruskal–Wallis ANOVA; *p* = 0.03). Soleus mass was on average 30% lower (main effect (ME) of WB by 2-way ANOVA, *p* = 0.003; data not shown) in both G/6 groups vs. WB controls after 21 days of partial weightbearing, verifying the efficacy of the G/6 treatment. Some animals were terminated immediately following 21 days of G/6, while some animals were allowed to recover at full weightbearing (normal cage activity) for 21 days following 21 days of G/6. There were no differences in body mass among groups following 21 days of recovery.

**Table 1:** Body mass following partial weightbearing ± radiation exposure for 21 days (post partial weightbearing) and after 21 additional days to assess long-term response (post recovery)

	WB-Sham	WB-Rad	G/6-Sham	G/6-Rad
Post-G/6 (g)	23.23 ± 2.27	23.31 ± 1.71	21.24 ± 1.25	20.74 ± 1.0
Post recovery (g)	23.98 ± 1.26	23.63 ± 1.88	23.69 ± 1.20	23.99 ± 1.15

Mice were exposed to 0.5 Gy ^56^Fe and/or G/6 for 21 days; recovery groups were returned to normal cage activity for 21 days after completing the initial 21 days of G/6 and/or radiation exposure. *WB* weightbearing, *Sham* no exposure to radiation, *Rad* acute dose of 0.5 Gy ^56^Fe, *G/6* partial weightbearing at 1/6 BW. Group means that share the same letter are not statistically different from one another (*p* < 0.05), as evaluated by Kruskal–Wallis ANOVA (data not normally distributed)

### Radiation led to improved cancellous bone mass and microarchitecture in the distal femur after 21 days, persisting through the recovery period

**Fig. 1:**
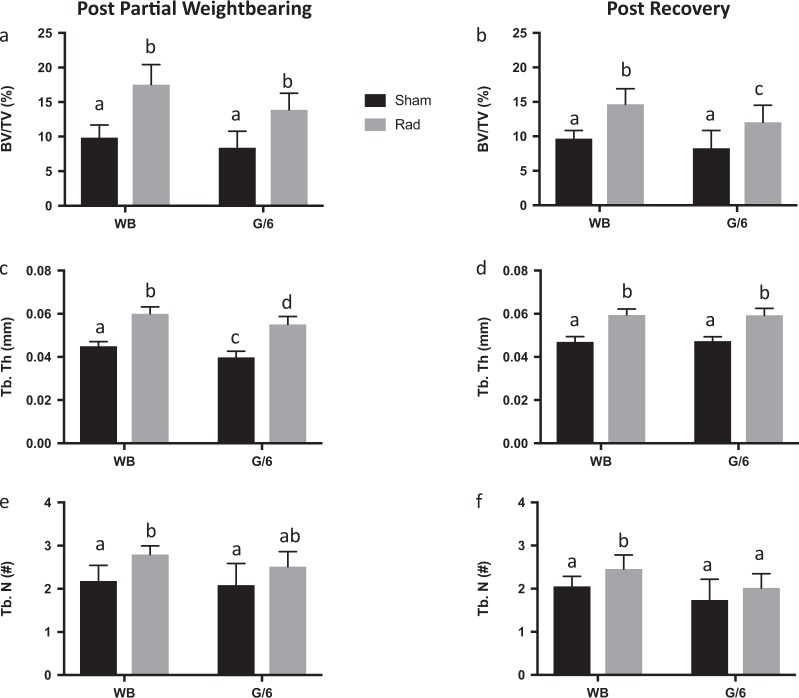
Distal femur cancellous bone mass and microarchitecture. Microcomputed tomography scans were performed on excised bone following exposure to 0.5 Gy ^56^Fe and/or 21 days of partial weightbearing at 1/6 g (G/6) (**a**, **c**, **e**) and following 21 days of recovery (**b**, **d**, **f**). **a**, **b** Percent bone volume per total volume (%BV/TV); **c**, **d** Trabecular thickness (Tb. Th); **e**, **f** Trabecular number (Tb. N). Sham-exposed animal means ± standard deviation are represented in black and Rad-exposed animal means ± standard deviation are represented in gray. Group means that share the same letter are not statistically different from one another (*p* < 0.05)

Twenty-one days after exposure to 0.5 Gy ^56^Fe, cancellous bone volume (%BV/TV) of the distal femur was 78% and 65% greater in WB-Rad and G/6-Rad groups, respectively, compared to their sham controls (Fig. 1; ME of radiation by 2-way ANOVA, *p* = 0.0001). This effect was due to a 33–38% greater trabecular thickness (Tb. Th) and 21–28% greater trabecular number (Tb. N) in the Rad mice vs. Sham mice (ME of radiation by 2-way ANOVA, *p* = 0.0001 and *p* = 0.002, respectively). This positive impact of acute high-LET radiation exposure was still evident after 21 days of weightbearing recovery (and 46 days after exposure) for %BV/TV, Tb. Th and Tb. N (Fig. 1; ME of radiation by 2-way ANOVA, *p* < 0.0001, *p* < 0.0001, and *p* = 0.01, respectively).

There was also a smaller but significant ME of G/6 (by 2-way ANOVA, *p* = 0.001) on distal femur cancellous trabecular thickness, which was 8–11% lower in sham-exposed G/6 animals compared with their WB controls after 21 days of partial weightbearing. There were no other MEs of G/6 at this bone site; after 21 days of weightbearing recovery, Tb. Th recovered to WB control values. L4 spine %BV/TV and Tb. N mean values were not different among groups (data not shown) after 21 days of G/6. Trabecular thickness at the L4 vertebral body was impacted by both G/6 and radiation (ME by 2-way ANOVA *p* = 0.025 and *p* = 0.001, respectively); a significant interaction (*p* = 0.001) for the two treatments is reflected in an 18% lower Tb. Th in sham-exposed but not irradiated G/6 mice. Mean values for Tb. Th in WB-Sham, WB-Rad, G/6-Sham, and G/6-Rad were 0.059 ± 0.006, 0.060 ± 0.004, 0.051 ± 0.005, 0.062 ± 0.004 mm, respectively. L4 samples were not collected at the post-recovery time point.

### Femoral cortical bone energy absorption and L4 ultimate load and stiffness improved with radiation exposure, but only in weightbearing mice

**Fig. 2:**
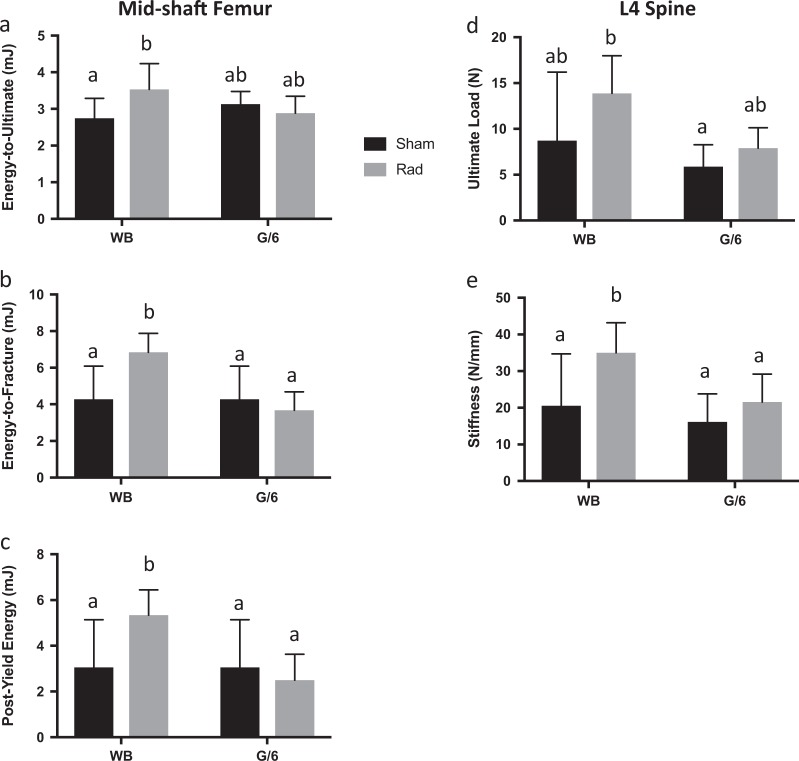
Cortical bone energy absorption and L4 vertebral structural properties. Mid-shaft femur energy absorption tested in three-point bending to failure (**a**–**c**) and L4 structural properties tested in compression (**d**–**e**) after 21 days of PWB and exposure to 0.5 Gy ^56^Fe. Sham-exposed animal means ± standard deviation are represented in black and Rad-exposed animal means ± standard deviation are represented in gray. Group means that share the same letter are not statistically different from one another (*p* < 0.05)

Mechanical testing was performed on the midshaft femur by three-point bending to failure and on the femoral neck with axial compression testing at both time points; L4 vertebral bodies collected after 21 days of G/6 and/or radiation were tested in compression (specimens were not available after 21 more days of weightbearing recovery). Femora in weight-bearing mice previously exposed to 0.5 Gy ^56^Fe exhibited no change in ultimate load or stiffness in bending (Table 2) at the 21-day time point; however, after 21 days of recovery stiffness was 20% greater in G/6 mice exposed to radiation vs. sham-exposed G/6 controls (Table 2; Kruskal–Wallis ANOVA, pairwise comparison *p* = 0.022). Energy absorption in bending was increased after 21 days as compared to sham-exposed controls (Fig. 2). A significant interaction (by 2-way ANOVA, *p* = 0.028) between G/6 and radiation effects reflects a 29% greater energy to ultimate load in WB-Rad vs. WB-Sham mice, but no differences between the two G/6 groups of mice. WB-Rad had a greater energy to fracture (ME of radiation by 2-way ANOVA, *p* = 0.039) and postyield energy (ME of G/6 by 2-way ANOVA, *p* = 0.008; interaction *p* = 0.019) than all other groups. The increase in energy absorption characteristics appears to be a transient effect, since there were no group differences between WB-Sham and WB-Rad mice for these variables after 21 days of recovery (Table 2).

**Table 2:** Bone mechanical properties following partial weightbearing ± radiation exposure for 21 days (post partial weightbearing) and after 21 additional days to assess long-term response (post recovery)

	WB-Sham	WB-Rad	G/6-Sham	G/6-Rad
*Post partial weightbearing*				
Mid-shaft Femur 3-point Bend				
Ultimate load (N)	18.6 ± 2.50	19.8 ± 1.90	19.6 ± 2.50	17.4 ± 2.10
Stiffness (N/mm)	91.6 ± 2.80	94.7 ± 14.90	101 ± 19.8	97.0 ± 18.3
Femoral neck compression				
Ultimate load (N)	11.2 ± 1.80	11.4 ± 1.30	10.0 ± 1.50	11.1 ± 0.80
Stiffness (N/mm)	28.9 ± 7.90	30.5 ± 12.2	23.0 ± 7.30	31.4 ± 7.23
*Post recovery*				
Mid-shaft Femur 3-point Bend				
Ultimate load (N)	20.5 ± 1.43	22.1 ± 2.13	21.7 ± 1.70	19.6 ± 1.80
Stiffness (N/mm)	89.0 ± 8.9	95.4 ± 36.6	79.5 ± 18.5	95.7 ± 26.5
Energy-to-ultimate (mJ)	3.05 ± 0.63	3.02 ± 0.06	3.32 ± 0.43	3.34 ± 0.92
Energy-to-fracture (mJ)	6.65 ± 1.60	4.79 ± 0.30	5.20 ± 1.24	4.66 ± 1.74
Postyield energy (mJ)	5.40 ± 1.73	3.33 ± 0.18	3.84 ± 1.33	3.35 ± 1.61
Femoral neck compression				
Ultimate load (N)	11.1 ± 1.75	12.0 ± 1.82	10.7 ± 1.79	11.4 ± 0.7
Stiffness (N/mm)	33.8 ± 12.1	25.3 ± 3.85	25.7 ± 6.50	29.1 ± 12.2

Two different mechanical tests are represented following 0.5 Gy ^56^Fe and/or G/6 for 21 days (Post partial weightbearing) and for separate groups maintained for 21 additional days of full weightbearing recovery (Post recovery). Mid -shaft femurs were subjected to three-point bending to failure and femoral necks to axial compression with quasi-static loading rates. *WB* weightbearing, *Sham* no exposure to radiation, *Rad* acute dose of 0.5 Gy ^56^Fe, *G/6* partial weightbearing. Group means that share the same letter are not statistically different from one another (*p* < 0.05); pairwise comparisons generated from Kruskal–Wallis ANOVA (data not normally distributed)

A positive impact of ^56^Fe exposure was observed in L4 vertebral body structural properties in weightbearing, but not G/6, mice after the initial 21 days. L4 stiffness was 70% greater in WB-Rad compared to WB-Sham (ME of radiation, by 2-way ANOVA *p* = 0.029); the higher value for ultimate load for WB-Rad mice was not statistically different from the WB-Sham values (Fig. 2). There was no evidence for any impact of radiation on femoral neck structural properties at either time point.

Reduced weightbearing (G/6) had a smaller impact on femoral mechanical properties than did high-LET radiation exposure. There were MEs of G/6 after 21 days, reducing energy to fracture (by 2-way ANOVA, *p* = 0.002) and postyield energy (by 2-way ANOVA, *p* = 0.008) (Fig. 2). Larger effects were observed in L4 vertebral body structural mechanical properties. Ultimate load and stiffness were 39% and 32% lower, respectively, in pooled G/6 groups vs. pooled WB groups (ME of G/6 by 2-way ANOVA, *p* = 0.039 and *p* = 0.048, respectively). No impact of previous partial weightbearing was detected after 21 days of recovery for any mechanical property at all bone sites tested.

### G/6 led to a decrease in midshaft tibia endocortical mineralizing surface while cancellous MAR was reduced after 21 days of weightbearing recovery

All mice had some fluorochrome labeling on cortical and/or cancellous bone surfaces (BSs), verifying delivery of labels. There are no significant pairwise comparisons in % mineralizing surface (%MS/BS), mineral apposition rate (MAR) and bone formation rate (BFR) of the distal femur cancellous bone among groups after 21 days of G/6 and/or radiation exposure. While not statistically significant, there were approximately two and threefold increases in distal femur cancellous %MS/BS in WB-Rad and G/6-Rad groups compared to corresponding sham groups. There were too few samples exhibiting double labels (at most, one animal per group) on the periosteal and endocortical surfaces of the mid-shaft tibia to determine MAR, without which BFR could not be calculated for these surfaces. No significant differences were detected in periosteal tibial %mineralizing surface. However, tibial endocortical %MS/BS was 74% lower in pooled G/6 groups compared to pooled WB groups (Table 3; ME for G/6 by 2-way ANOVA, *p* = 0.001).

**Table 3:** Histomorphometric measures of bone formation following partial weightbearing ± radiation exposure for 21 days (post partial weightbearing) and after 21 additional days to assess long-term response (post recovery)

	WB-Sham	WB-Rad	G/6-Sham	G/6-Rad
*Post partial weightbearing*				
Distal femur				
MS/BS (%)	5.94 ± 4.83	13.71 ± 6.28	2.61 ± 3.31	8.59 ± 3.22
Double Label Present	6 of 6	5 of 5	6 of 8	4 of 4
MAR (um/d)	0.62 ± 0.37	0.63 ± 0.41	0.14 ± 0.13	2.18 ± 2.70
BFR (um³/um²/y)	9.57 ± 8.75	37.4 ± 29.1	1.07 ± 1.17	83.1 ± 107
Midshaft tibia				
Endocortical MS/BS (%)	22.20 ± 13.39	23.35 ± 12.89	7.30 ± 2.49	4.68 ± 5.82
Double Label Present	1 of 4	1 of 7	0 of 5	0 of 7
Periosteal MS/BS (%)	2.24 ± 3.49	2.19 ± 2.09	1.12 ± 1.6	0.79 ± 1.02
Double Label Present	1 of 4	0 of 7	0 of 5	0 of 7
*Post recovery*				
Distal femur				
MS/BS (%)	16.0 ± 3.49	18.9 ± 6.18	19.74 ± 9.34	15.60 ± 5.60
Double Label Present	4 of 5	4 of 4	8 of 9	7 of 7
MAR (um/d)	1.97 ± 0.74	1.50 ± 1.02	1.33 ± 0.40	0.79 ± 0.49
BFR (um³/um²/y)	85.9 ± 52.1	110 ± 89.6	78.4 ± 56.8	50.3 ± 34.7
Midshaft tibia				
Endocortical MS/BS (%)	14.17 ± 9.49	18.07 ± 11.65	32.06 ± 15.34	37.5 ± 11.1
Double Label Present	3 of 7	2 of 6	3 of 6	4 of 7
Periosteal MS/BS (%)	14.38 ± 7.71	6.66 ± 4.2	18.61 ± 11.86	13.5 ± 7.92
Double Label Present	4 of 7	1 of 6	3 of 6	2 of 7

Mice were exposed to 0.5 Gy ^56^Fe and/or G/6 for 21 days; recovery groups were returned to normal cage activity for 21 days after completing the initial 21 days of G/6 and/or radiation exposure. All mice received two calcein injections in the week preceding end of each experiment. Distal femur cancellous bone in ~ 1 mm^2^ area was assessed, as well as periosteal and endocortical surfaces at the midshaft tibia. Proportion of samples with double label present are reported for each bone site. *WB* weightbearing, *Sham* no exposure to radiation, Rad acute dose of 0.5 Gy ^56^Fe, *G/6* partial weightbearing, *MS/BS* mineralized surface relative to total bone surface, *MAR* mineral apposition rate, *BFR* bone formation rate. Group means that share the same letter are not statistically different from one another (*p* < 0.05); pairwise comparisons generated by 2-way ANOVA and Duncan’s post hoc testing

Following recovery, cancellous %MS/BS was not different among groups (Table 3); however, distal femur cancellous MAR, indicative of focal osteoblast activity, was 39% lower in pooled G/6 vs. pooled WB groups (ME of G/6 by 2-way ANOVA, *p* = 0.001, Table 3). There were no differences among groups in cancellous BFR. Too few samples exhibited double labels on the periosteal and endocortical surfaces of the mid-shaft tibia to determine MAR after recovery; therefore, BFR could not be calculated for these surface. There were no relevant differences in %MS/BS at this time point on either the endocortical or periosteal surface.

## Discussion

The primary findings of these experiments are (1) radiation, contrary to our initial hypothesis, resulted in improved cancellous bone microarchitecture in both WB and G/6 mice and improved energy absorption characteristics in the femur of WB mice; (2) there were site-specific responses to radiation in the distal femur and lumbar vertebra, with a greater impact of radiation on the cancellous bone of the distal femur than that of L4; and (3) after recovery (~45 days after irradiation), the positive impact of radiation on cancellous microarchitecture of the distal femur was maintained, but this did not hold true for mid-shaft femur energy absorption.

Most previous studies assessing the effects of space-relevant radiation on bone have found decrements in bone volume and structural quality following exposure.^13–19^ Much of the previous literature examines radiation doses higher than those examined in this study.^13,20–22^ Fewer studies have investigated the effects of lower doses of high-LET radiation (e.g., 0.5 Gy ^56^Fe) on bone. One such study found no effect of 0.5 Gy of ^56^Fe radiation on L4 vertebral bone parameters when measured 3 days after exposure in weightbearing C57BL/6J adult mice.^13^ Our previous investigation documented nonsignificantly lower %BV/TV and significantly lower trabecular number (−15%) at the distal femur after exposure to an acute and/or fractionated 0.5 Gy dose of Si in weightbearing BALB/cByJ adult mice when assessed 21 days after exposure.^11^ By contrast, a positive impact of very low-dose radiation has been reported on distal femur cancellous bone in C57BL/6J adult mice who were exposed to collimated head-only iron-ion ^56^Fe dose with a collimator interposed between the beam and the animals, resulting in a 0.04 Gy exposure of the hindlimbs to a field of secondary particles including protons, ^4^He and neutrons. Nearly a year later (11.5 months), irradiated mice exhibited a 51% greater cancellous %BV/TV in the distal femur.^23^ Given this study’s design, it is possible that some of the changes observed in tissues distant to the brain might have been influenced by radiation impact on the CNS. In the current study conducted over a much shorter time span, we found that a whole-body 0.5 Gy ^56^Fe exposure led to greater (+65–78%) distal femur cancellous bone volume in irradiated mice vs. sham controls when measured 25 days after exposure, with significant increases in both trabecular number and thickness; these improvements in indices of cancellous bone microarchitecture with radiation exposure largely persisted for up to 46 days post-radiation, consistent with the findings of Karim and Judex^23^ but at a dose ten-fold higher and over a much shorter time frame.

The mechanisms explaining positive effects of low-dose radiation on bone cell activity and cancellous microarchitecture are not well-defined. Most published studies (often at higher doses than 0.5 Gy) demonstrate an increase in osteoclast activity, driving bone resorption, and/or a decrease in osteoblast-driven bone formation following radiation exposure, resulting in a net loss of bone.^15,20,21,24,25^ In the current study, we document a two to threefold larger %MS/BS, an important indicator of numbers of active osteoblast teams, in distal femur cancellous bone of both WB and G/6 irradiated mice. There was large variability in this response, resulting in statistically non-significant comparisons. An increase in osteoblast activity would be consistent with the large (33–38%) increases observed in trabecular thickness at this bone site in mice exposed to an acute ^56^Fe dose 25 days earlier. In a very different challenge to bone homeostasis, closed femoral fractures in young adult male rats exposed to a 0.5 Gy X-ray dose exhibit accelerated fracture healing, with more rapid completion of endochondral and intramembranous bone formation at the fracture callus site.^26^ In vitro experiments offer some clues to mechanisms. When preosteoblastic cells are exposed to 0.5 Gy X-ray radiation, molecular markers of osteoblast differentiation exhibit a short-term increase at 7 days post-exposure^26^ In primary cell cultures derived from healthy C57Bl/6 mice and exposed to acute doses of low-dose X-ray radiation (0.1–1.0 Gy), osteoclast resorbing activity (pit area) decreased while osteoblast activity (mineralizing area) increased. The anti-inflammatory cytokine transforming growth factor-beta (TGF-beta), which stimulates osteoblast activity, and secreted osteoprotogerin (OPG), a downregulator of osteoclastogenesis, increased in cultures of fibroblast-like synoviocytes (derived from cartilage) at 96 h after the 0.5 Gy X-ray exposure.^27^

Previous studies of the impact of partial weightbearing on bone integrity have demonstrated decrements even when mice are maintained at 70% of full weightbearing.^8,10,11^ If mechanical loads are reduced to 20% of normal weightbearing, losses in cancellous bone volume are as severe as that seen with full unloading (traditional tail suspension).^8^ Our research group has previously reported significant losses in cancellous bone mass (9–13%) and BFR (46–54%) in G/6 mice (~17% weightbearing).^11,12^ In the current study, we report no statistically significant decrements in %BV/TV at the distal femur or L4 vertebra sites; deficits only in trabecular thickness at both sites were observed after 21 days of G/6. Mineralizing activity (%MS/BS) at the mid-shaft tibial endocortical surface was reduced three to fourfold in G/6 mice after 21 days of partial weightbearing; reductions observed in the distal femur cancellous bone compartment for %MS/BS, MAR and BFR did not reach statistical significance. Importantly, mice in the current study were allowed up to 4 days of recovery in full weightbearing (including transport from Brookhaven National Laboratory in New York to Texas) before beginning a period of one-sixth weightbearing, whereas in our previous work mice commenced the G/6 treatment within minutes of the ^28^Si exposures at the same location.^11^ It is possible that the stress of transport cross-country induced some preliminary loss of cancellous bone mass, diminishing the likelihood of detecting further losses with G/6 treatment. Sham-exposed WB controls in the previous study had %BV/TV values of 12–16%, whereas those same controls in the current study exhibited %BV/TV of just under 10%.^11^

Given that spaceflight travel outside of low Earth orbit for 3 years will expose human crew members to high-energy ionizing radiation, it is imperative to determine if there are interactive effects of prolonged mechanical unloading and radiation exposure. At higher doses, radiation exposure appears to exacerbate the negative effect of disuse in hindlimb unloaded rodents.^19,28^ Rats exposed to full unloading via tail suspension immediately after a 4 Gy X-ray exposure exhibit a synergistic loss of bone with the combined treatment.^28^ Likewise, a separate investigation documented an additive effect of 1 Gy proton irradiation (delivered 36 h before the initiation of hindlimb unloading) and disuse on loss of bone mass in 15-week-old female C57/Bl6 mice.^19^ At space-relevant radiation doses below 1 Gy,^7^ the impact on cancellous bone mass in combination with disuse is more variable. Our group previously documented that exposure to 0.5 Gy ^28^Si (either as one acute dose or 3 fractions of 0.17 Gy) exerted no additional impact on reductions in distal femur cancellous bone volume in 16-week-old female BALB/cByJ female mice maintained at 1/6 partial weightbearing, but did exacerbate reductions in endocortical %MS/BS.^11^ Interestingly, when a single dose of 0.5 Gy ^56^Fe is delivered 3 days prior to the end of a 7-day hindlimb unloading period, rapid cancellous bone loss occurs along with an increase in osteoclast number, suggesting that bone cells of 16-week-old male C57/Bl6 mice were sensitized to radiation by the preceding 4 days of zero weightbearing.^29^ A recently published study, unique in delivering 1.7 Gy gamma radiation at very low-dose rates continuously over 20 days to 14-week-old C57/Bl6 female mice, demonstrated no exacerbation of bone structural decrements with concurrent hindlimb unloading.^30^ Given that in the current study our high-LET radiation dose was delivered to ambulatory mice on day 1 of the experiment and the G/6 treatment began only 4 days later, it might be that local production of TGF-beta and OPG as observed by Deloch et al. in isolated bone/cartilage cells 96 h after irradiation contributed to the positive impacts on bone structure and, possibly, osteoblast function observed in our irradiated mice.^27^ Alternatively, it may be that the four days of normal ground reaction forces experienced immediately following high-LET exposure (before the G/6 treatment began) in the current study might have provided enough anabolic stimulus to counteract any early negative impact of an acute radiation exposure on osteoblast activity.

A strength of the current study is the comprehensive evaluation of the impact of G/6 and/or high-LET radiation exposure on bone mechanical properties, which have not been consistently evaluated in past studies. After 21 days of simulated Mars gravity conditions (3/8 g or 37% full weightbearing), femoral ultimate moment was 27% lower vs. aging controls; bending stiffness and moment at yield were also significantly lower.^10^ Alwood et al. found a 24% reduction in the compressive yield force in L4 vertebrae of adult mice exposed to both 0.5 Gy ^56^Fe radiation and hindlimb unloading; interestingly, the 35% reduction in L4 stiffness observed with unloading alone was not exacerbated by concurrent radiation exposure.^13^ In the current study, femora of weightbearing mice exposed to 0.5 Gy ^56^Fe exhibited 29% higher energy-to-ultimate load values and also had higher energy-to fracture and post-yield energy (from the yield to fracture point) values. These observations suggest that cortical bone ductility was increased by radiation exposure and might improve fracture resistance in certain scenarios. This positive effect of high-LET radiation exposure was not observed in G/6 mice, who exhibited energy absorption values similar to sham-exposed controls.

Our conclusions should be interpreted in the context of several limitations. We studied only one strain of mouse and one sex. Our choice of BALB/cByJ female mice was based primarily on previous success with this strain and sex in the inaugural partial weightbearing studies.^8^ The use of a radiosensitive species of mice (BALB/cByJ)^31^ does provide an increased probability of revealing potential differences in response to radiation exposure based on strain. Since we did not apply the moleskin jackets or suspension harness, we did not control for the slight additional stress imposed by this partial weightbearing model.^10^ Given that we collected tissues at only two time points, it is possible that we missed significant changes in the bone response that occurred early after radiation exposure or at the beginning of the partial weightbearing period. It would have been informative to have measures of osteoclast activity, but technical error reduced the availability of samples for histomorphometric analyses of osteoclast surface. Finally, the radiation in this experiment was delivered in one acute dose, the response to which might vary considerably from that to a very low-dose-rate, continuous exposure over an extended period of time, which would better simulate GCR exposures during future missions to Mars and the Moon.

Overall, our findings provide no evidence for worsening of disuse bone effects by radiation exposure. Rather, we found a persistent positive effect of high-LET radiation exposure on cancellous bone volume and trabecular thickness in both full- and partial-weightbearing adult mice, with the impact generally larger in magnitude at the distal femur vs. that observed in L4 vertebral bone. We also provide evidence for a short-term improvement in energy absorption characteristics of midshaft cortical bone. Cellular and molecular mechanisms for these positive impacts need to be identified. Previously published data document positive impacts of lower doses of radiation on cancellous bone microarchitecture^23^ and other tissues, e.g., angiogenesis in ischemic tissue^32^ and human fibroplast repair of higher dose radiation-generated chromosomal breaks.^33^ Our results confirm deleterious impacts of 1/6 weightbearing and provide new evidence for interesting positive effects of low-dose radiation on cancellous and cortical bone integrity.

## Methods

### Experimental design, animals, and radiation exposure

**Fig. 3:**
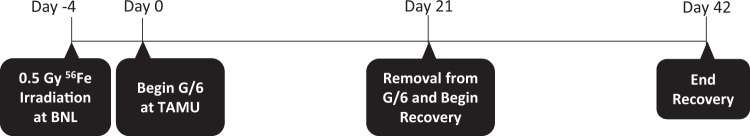
Experimental timeline for partial weightbearing ± radiation treatments and recovery therefrom. Animals were irradiated (0.5 Gy ^56^Fe) at Brookhaven National Laboratory and transported to Texas A&M University. Four days after exposure, half of the radiation-exposed animals were placed into G/6 while the rest remained weightbearing as controls (same for sham). After 21 days of G/6, half of the animals from radiation and sham groups were terminated. The remaining animals were allowed to recover in full weightbearing for 21 days

This study was approved by the Institutional Animal Use and Care Committees (IACUC) at Texas A&M University (College Station, TX) and Brookhaven National Laboratory (BNL: Upton, NY) and complies with all relevant ethical regulations. Young adult female BALB/cByJ mice (16 weeks old; Jackson Labs, Bar Harbor, ME; *n* = 62) were transported directly to BNL for radiation exposure. We chose to work with female mice to improve power of statistical analyses, given the limited number of mice that could be exposed at the BNL NASA Space Radiation Laboratory (NSRL) for any one experiment and to afford direct comparisons with previous studies on partial-weightbearing effects.^8,10,11^ Half of the mice were irradiated at the NSRL at a dose of 0.5 Gy of 1 GeV/nucleon ^56^Fe at a dose rate of 0.5 Gy/min; conscious animals were positioned 90° to the beam path in a 50 ml conical tube to achieve a whole-body exposure. Sham-irradiated mice were transported from the BNL Medical Department to the NASA Space Radiation Laboratory holding room along with the irradiated mice and were placed in the same conical tubes for the same duration (<12 min). Within 24 h, all mice were shipped overnight to the animal facility at Texas A&M University and allowed to acclimate in regular shoebox cages for 3 days. Four days following radiation exposure, animals were randomly assigned to experimental groups and half were placed into custom-made lucite partial weightbearing cages at 1/6 full body weight (G/6) for 21 days (Fig. 3a; G/6-Sham, *n* = 15 or G/6-Rad, *n* = 16). Weightbearing cage controls (WB) were singly housed in shoebox cages (WB-Sham, *n* = 14 or WB-Rad, *n* = 16). Half the mice in each group were terminated at 21 days and tissues collected. The remaining G/6 animals of each group were removed from suspension and allowed free cage activity for 21 days while singly housed in shoebox cages to assess long-term effects. Animals were maintained at a standard temperature (23 ± 2 °C) and a 12:12 h light-dark cycle throughout the experiment. Standard rodent chow (no. 8604; Harlan Teklad, Madison, WI) pellets were placed in a shallow dish on the floor of the cages; and water was available *ad libitum* via standard bottles mounted on the cage exterior, with spouts accessible via reinforced holes in the lucite cage walls. Animal health was monitored several times daily and body weight was recorded every other day.

### Partial weightbearing suspension

A horizontal partial weightbearing harness system was used to simulate Lunar conditions (one-sixth Earth’s gravity) in mice for 21 days as previously described.^10–12^ All mice (including controls) were singly housed in 13-in^3^ custom-built cages consisting of clear polycarbonate walls with removable polypropylene perforated floors, as previously described.^10^ Mice assigned to G/6 groups were suspended horizontally from a rod across the top of the cage with moleskin jackets at the shoulders and a SteriStrip (3 M, St. Paul, MN) at the base of the tail in order to reduce weightbearing of the forelimbs and hindlimbs equally, as previously described.^11,12^ To minimize animal stress, jackets and tail wraps were applied under isoflurane anesthesia. Control mice were not harnessed, nor were the moleskin jackets applied. Previous work did demonstrate some bone responses to being jacketed; however, those jacketed mice were singly housed, pair-fed to PWB mice, and compared to double-housed, ad-lib fed controls aging control, so it is impossible to assess which factor (jacketing, short-term caloric restriction, or housing condition) contributed to those differences.^10^ Full body weight of G/6 mice was measured every other day by suspending the whole mouse while attached to the suspension apparatus in a custom-made titration frame, built to precisely the same floor-to-rod height as the suspension cages, set atop an electronic scale (Ohaus Corp., Pine Brook, NJ). Weightbearing was titrated to one-sixth body mass ±0.1 g daily by adjusting the linear tension spring (spring constant of 0.7 N/m) connecting the suspended mouse to the roller on the cage-top rod.

### Euthanasia and tissue harvest

Calcein injections (15 mg/kg body weight, Sigma Aldrich, St. Louis, MO) to label mineralizing bone were given 9 and 2 days prior to termination. To ensure G/6 animals’ limbs did not bear full body weight prior to termination, all G/6 animals were anesthetized before removal from suspension apparatus with a cocktail of Ketaset and Dexdomitor (3:2 ratio, Ket:Med) and then euthanized by thoracotomy and exsanguination via cardiac puncture. The lumbar spine and the left femur were wrapped in phosphate-buffered saline solution (PBS) soaked, covered with additional PBS and stored at −20 °C. The distal tibia and distal femur were fixed in 10% phosphate-buffered formalin for 12 h, then stored at 4 °C in 70% ethanol for microcomputed tomography (µCT_ and histomorphometry. Lumbar spine was not collected at the recovery timepoint due to minimal changes observed after first 21 days.

### Evaluation of cancellous microarchitecture by µCT

The left femur and 4th lumbar vertebra from each animal were assessed by µCT on a Skyscan^™^ 1172 (SkyScan, Kontich, Belgium). X-ray acquisition settings were set at 55 kVp and 145 lA, with an integration time of 200 ms. Scans were performed with an isotropic voxel size of 6 µm and images were reconstructed in 1024 × 1024 pixel matrices. A global threshold of 35% maximal gray scale was determined by visual inspection. Structural parameters were measured using standard guidelines and protocols.^34^ Key outcomes reported in both the femur and the spine are bone volume normalized to tissue volume (%BV/TV) and trabecular thickness (Tb. Th), number (Tb. N) and spacing (Tb. Sp).

### Mechanical properties

All mechanical properties testing was conducted on an Instron 3345 machine (Norwood, MA; 100N load cell; Bluehill v. 2.14.582). Load–displacement data were recorded at 10 Hz and analyzed using Matlab (The Mathworks, Inc.; Natick, MA). Stiffness (k, N/mm) was determined by calculating the slope of the load–displacement curve in the elastic region. Ultimate load (UL, N) designated the largest force achieved throughout the test. Data were processed post hoc using a custom-written Matlab program (version 7.12.0, The MathWorks, Inc.) to generate outcomes using force–displacement curves described below. To assess cortical bone mechanical properties, three-point bending to failure was performed on the femoral diaphysis.

With the femur positioned anterior side down resting on two 2-mm diameter metal pin supports spaced 10 mm apart, loading was applied through an upper pin that contacted the posterior surface at mid-shaft (50% total bone length). A quasi-static loading rate (2.54 mm/min) was used and load applied until fracture occurred. Load cell displacements were monitored and recorded using a linear variable differential transformer. In addition to the extrinsic, or whole bone, properties of stiffness and ultimate load, various energy-absorbed parameters were determined as the area under the load–displacement curve: energy-to-ultimate load (mJ), energy-to-fracture (mJ), and post-yield energy (mJ), from yield point to fracture.

Femoral neck strength was evaluated using the proximal half of the femur generated after three-point bending was completed. Each proximal femur was stabilized vertically with the femur shaft firmly inserted into a metal support plate. Quasi-static load (2.54 mm/min) was applied to the femoral head parallel to the femoral shaft long axis until fracture occurred. Stiffness (N/mm) and ultimate load (*N*) were derived as detailed above. Lumbar vertebrae were tested in uniaxial compression to determine the strength of the entire vertebral body, both cortical, and cancellous regions. Following the approach of Tommasini et al.^35^, L4 vertebrae were minimally shaved flat with a scalpel blade on cephalic and caudal endplates to achieve parallel testing surfaces. To ensure that the vertebra remained stationary during compression testing, a thin layer of epoxy was applied to the platens and an alignment pin was attached to the lower platen and placed through the vertebral foramen. Axial compression was applied to the caudal surface of the vertebral body by a 3-mm diameter platen at a rate of 0.05 mm/s. Stiffness (N/mm) and ultimate load (N) were determined as detailed above.

### Cortical histomorphometry

Undemineralized distal tibia were serially dehydrated and embedded in methyl-methacrylate (J.T. Baker, VWR, Radnor, PA). Serial cross-sections (100 µm thick) were cut using a diamond wafer low-speed saw (Buehler, Lake Bluff, IL) starting 1 mm proximal to the tibia–fibular junction. Image analysis was performed with an epifluorescent light microscope interfaced with a CCD video camera (Model, company) and OsteoMeasure Analysis Software, V 3.3 (OsteoMetrics, Atlanta, GA) to measure single- (sLS) and double-labeled (dLS), and total bone (BS) surfaces in order to compute mineralizing surface (%MS/BS = [(sLS/2) + dLS]*100/BS) on periosteal and endocortical surfaces at ×200 magnification. MAR = interlabel width/7 d; µm/d, and BFR = %MS/BS × MAR, μm^3^/μm^2^/year are not reported for cortical bone outcomes because less than 50% of samples in each group contained double label, disallowing computation of these variables.

### Cancellous histomorphometry

Undemineralized distal femurs were serially dehydrated and embedded in methyl methacrylate (J.T. Baker, VWR, Radnor, PA). Serial frontal sections were cut 8-μm thick using a motorized microtome (Leica; city state) and left unstained for fluorochrome label measurements. A defined region of interest was established starting 400 μm from the growth plate and within the endocortical edges encompassing approximately 1 mm^2^. Total BS, sLS, dLS, and interlabel distances were measured at ×200 magnification. MAR, µm/d, %MS/BS, and computed BFR (BFR = %MS/BS × MAR × 360, µm^3^/µm^2^/year) were calculated. Histomorphometric analyses and outcomes reported followed standardized methods and terminology.^36^

### Statistical analysis

All data are presented as mean ± standard deviation of the mean (SD) and were evaluated for differences using SPSS (IBM, version 23). Outliers were detected and removed by Grubbs test, a studentized outlier test; this resulted in the removal of only 4 data points across all outcomes (see Supplementary Table 1). A Shapiro–Wilk test for normality was conducted; those measures not normally distributed were subjected to nonparametric testing. A two-factor ANOVA was used for all normally distributed data in this paper; significant main and interaction effects of the variables within the test are reported for these analyses. Post hoc pairwise comparisons were made using Duncan’s test. For groups with data not normally distributed, a Kruskal–Wallis ANOVA was performed with significant pairwise comparisons reported; MEs for these tests cannot be reported. Data are reported as means ± standard deviations. Significance was accepted at *p* < 0.05.

### Reporting summary

Further information on research design is available in the Nature Research Reporting Summary linked to this article.

## Supplementary information

Supplementary Table 1.

REPORTING SUMMARY FORM

## Data Availability

The data sets generated during and/or analyzed during the current study are available from the corresponding author on reasonable request.

## Supplementary information

**Supplementary Information** accompanies the paper on the *npj Microgravity* website (10.1038/s41526-019-0074-3).
