# Supplementary material for: Positive impact of low-dose, high-energy radiation on bone in partial- and/or full-weightbearing mice
Source: NPJ Microgravity. 2019 Jun 4;5:13. doi: 10.1038/s41526-019-0074-3 (PMC6547738; doi:10.1038/s41526-019-0074-3)
Supplement: Supplementary file 1 — Supplementary Table 1. [file 41526_2019_74_MOESM1_ESM.pdf]

Supplementary Table 1. **Tally of samples numbers for each outcome variable + number of outliers detected/excluded (Grubbs test)**

		WB-Sham		WB-Rad		G/6-Sham		G/6-Rad	
**Location/Test**	**Measure**	**Number of Samples Assessed**	**Outliers**	**Number of Samples Assessed**	**Outliers**	**Number of Samples Assessed**	**Outliers**	**Number of Samples Assessed**	**Outliers**
Whole body	Body Mass	5		9		5		7	
Whole body	L. Soleus Mass	9		9		8		8	
Whole body	R. Soleus Mass	9		9		8		8	
Distal Femur	Percent Bone Volume (%)	5		8		5		8	
Distal Femur	Trabecular Thickness (mm)	5		8		5		8	
Distal Femur	Trabecular Number (#)	5		8		5		8	
L4 Spine	Percent Bone Volume (%)	8		7		8		8	
L4 Spine	Trabecular Thickness (mm)	8		7		8		8	
L4 Spine	Trabecular Number (#)	8		7		8		8	
Mid Shaft Femur 3 point bend	Ultimate Load (N)	5		8		5		7	1
Mid Shaft Femur 3 point bend	Stiffness (N/mm)	4	1	8		5		7	1
Mid Shaft Femur 3 point bend	Energy-to-Ultimate (mJ)	5		8		5		8	
Mid Shaft Femur 3 point bend	Energy-to-Fracture (mJ)	5		8		5		8	
Mid Shaft Femur 3 point bend	Post-yield Energy (mJ)	5		8		5		8	
Femoral Neck Compression Test	Ultimate Load (N)	7		8		7		7	
Femoral Neck Compression Test	Stiffness (N/mm)	7		8		7		7	
L4 Compression Test	Ultimate Load (N)	6		6		7		4	
L4 Compression Test	Stiffness (N/mm)	6		6		7		4	
Distal Femur	MS/BS (%)	6		5		8		4	
Distal Femur	MAR (um/d)	6		5		8		4	
Distal Femur	BFR ($um^3/um^2/y$)	6		5		8		4	
Mid Shaft Tibia	Endocortical MS/BS (%)	4		7		5		7	
Mid Shaft Tibia	Periosteal MS/BS (%)	3	1	7		5		7	
